# Adaptive social immunity in leaf-cutting ants

**DOI:** 10.1098/rsbl.2009.0107

**Published:** 2009-05-01

**Authors:** Tom N. Walker, William O. H. Hughes

**Affiliations:** Institute of Integrative and Comparative Biology, University of Leeds, Leeds LS2 9JT, UK

**Keywords:** parasite, social insect, grooming

## Abstract

Social insects have evolved a suite of sophisticated defences against parasites. In addition to the individual physiological immune response, social insects also express ‘social immunity’ consisting of group-level defences and behaviours that include allogrooming. Here we investigate whether the social immune response of the leaf-cutting ant *Acromyrmex echinatior* reacts adaptively to the virulent fungal parasite, *Metarhizium anisopliae*. We ‘immunized’ mini-nests of the ants by exposing them twice to the parasite and then compared their social immune response with that of naive mini-nests that had not been experimentally exposed to the parasite. Ants allogroomed individuals exposed to the parasite, doing this both for those freshly treated with the parasite, which were infectious but not yet infected, and for those treated 2 days previously, which were already infected but no longer infectious. We found that ants exposed to the parasite received more allogrooming in immunized mini-nests than in naive mini-nests. This increased the survival of the freshly treated ants, but not those that were already infected. The results thus indicate that the social immune response of this leaf-cutting ant is adaptive, with the group exhibiting a greater and more effective response to a parasite that it has previously been exposed to.

## 1. Introduction

Parasites may be a particularly significant threat for social insects because their colonies normally contain dense aggregations of highly related individuals (Schmid-Hempel 1998; Boomsma *et al*. 2005). Just as in other animals, social insects have individual-level defences against parasites, such as the physiological immune response. Unlike solitary animals, however, social insects also have group-level defences. These include allogrooming, a behaviour that is effective at removing parasites and increasing resistance (Rosengaus *et al*. 1998; Hughes *et al*. 2002; Yanagawa *et al*. 2008). Such group-level defences can be regarded as a form of ‘social immunity’ (Cremer *et al*. 2007; Cremer & Sixt 2009). The social immune response may even be transferable, with the resistance of naive individuals being increased by interacting with individuals that have been exposed to a parasite (Traniello *et al*. 2002; Ugelvig & Cremer 2007).

Insects are well known to be able to learn tasks and the stimuli associated with them, increasing their behavioural efficiency with experience (Leadbeater & Chittka 2007). It is therefore possible that the social immune response of social insects may be adaptive and improve when the group responds to a disease threat against which it has been ‘immunized’ by previous exposure. Here we use mini-nests of the leaf-cutting ant *Acromyrmex echinatior* to examine whether their social immune response has such an adaptive component.

## 2. Material and methods

Six colonies of *A*. *echinatior* were used, all collected from Gamboa, Panama. The parasite used was strain KVL02-73 of *Metarhizium anisopliae* var. *anisopliae*, which came from the same site (Hughes *et al*. 2004*b*). Highly pathogenic to *A*. *echinatior*, it kills ants dose dependently within as little as 3 days of exposure (Hughes *et al*. 2002, 2004*a*). Mini-nests consisted of a box (6 × 4 × 2 cm), two-thirds filled with the mutualist fungus and containing two large larvae, placed within a lidded arena (10 cm diameter × 6 cm height). Each mini-nest was given 15 small workers (<1.4 mm head width), 4 intermediate-sized workers (1.4–1.8 mm head width) and 2 large workers (>1.8 mm head width). Mini-nests were supplied with water and 10 per cent sugar water ad libitum.

Six mini-nests were set up for each of the six colonies. Half were used as immunized mini-nests. These mini-nests each received two nest-mate cadavers sporulating with *Metarhizium*, two live nest-mates treated 2 days previously with *Metarhizium* and two live nest-mates treated with *Metarhizium* immediately before introduction to the mini-nest. This combination ensured significant exposure because although sporulating cadavers carried approximately 2 × 10^6^ conidia, they were interacted with very little by other ants, whereas the live ants carried only 5 × 10^3^ conidia but were interacted with very frequently. The other mini-nests were kept naive with respect to *Metarhizium*, receiving nest-mates treated with a control solution of 0.05 per cent Triton-X in the same numbers as the immunized mini-nests, i.e. two ants freshly treated, two treated 2 days previously and two killed by freezing. Mini-nests were checked and dead ants removed daily for 14 days. The introduced cadavers were removed at the end of this period. The exposure procedure was then repeated and the mini-nests monitored for a further 14 days. Additional ants were added to each mini-nest from the source colony at the start and end of the second exposure to replace those that had died (*ca* 20% on average both in immunized and naive) and thus standardize the numbers of ants in each mini-nest at these points.

Two weeks after the second exposure, the social immunity of the mini-nests was assessed. Six intermediate-sized workers from each colony had 0.5 µl of 1 × 10^6^ *Metarhizium* spores (conidia)/ml suspension applied to their thorax (Hughes *et al*. 2002, 2004*a*) and left in isolation for 2 days. *Metarhizium* conidia germinate and penetrate the cuticle of the host insect within 24–48 h (Boucias & Pendland 1998; W. O. H. Hughes 2006, unpublished data); after 2 days, therefore, these ants were infected but no longer infectious, because all viable *Metarhizium* conidia on their cuticle would have germinated within this period. A second set of six intermediate-sized workers from each colony were also treated with *Metarhizium* and used immediately. At this time, therefore, these ants were not infected, but were infectious, as they carried viable *Metarhizium* conidia that could potentially infect other ants on their cuticles. As controls, the same numbers of ants were treated with 0.5 µl of 0.05 per cent Triton-X. Ants were paint-marked to allow recognition. One ant from each treatment was placed within each mini-nest from its colony in a random order at 30 min intervals. The mini-nest was observed for 30 s at 0, 2, 6, 8, 10, 20 and 30 min after each ant was added. The frequency with which treated ants were antennated or allogroomed by other ants in the mini-nests, as well as the frequencies of self-grooming by untreated ants, was recorded. Dead ants were then recorded and removed for 14 days. The effects of treatment (*Metarhizium* or control), time between treatment and placement in the mini-nest (0 or 2 days) and immunization (immunized or naive) on ant behaviour were examined using repeated-measures analysis of variance. The effects on ant mortality during the final 14 day assessment were examined using the Cox proportional hazards regression model. Treatments were compared pairwise using Kaplan–Meier survival analysis with the Breslow statistic, using *q*-values to control the false discovery rate (Storey & Tibshirani 2003). The numbers of non-focal ants surviving in immunized and naive mini-nests at the end of the final assessment were log transformed and compared using a *t*-test.

## 3. Results

**Figure 1.**
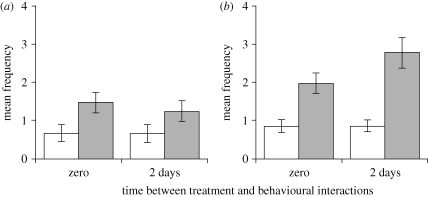
Mean ± s.e. frequency with which ants treated with either the fungal parasite *Metarhizium* (shaded columns) or a control solution (clear columns) were allogroomed for (*a*) 18 naive mini-nests that had not previously had contact with the parasite, and (*b*) 18 immunized mini-nests that had previously been exposed to the parasite. Ants were placed in mini-nests for behavioural observation either immediately after treatment or 2 days after treatment. Both naive and immunized mini-nests consisted of three mini-nests from each of six colonies.

The colony of origin did not affect either the behaviour or survival of ants (*p* > 0.05 in all cases). In both immunized and naive mini-nests, *Metarhizium*-treated ants received significantly more allogrooming than control-treated ants (immunization × treatment interaction: F_3,102_ = 1.18, *p* = 0.322; treatment: F_3,102_ = 3.4, *p* = 0.021; figure 1). This was true for ants treated 2 days, as well as immediately, before introduction (figure 1). Most allogrooming was carried out by intermediate-sized workers (F_2,102_ = 1.13, *p* < 0.0001). Immunized ants allogroomed more than ants in naive mini-nests (F_1,34_ = 4.46, *p* = 0.042; figure 1). Immunization had no effect on the frequency of antennation (F_1,34_ = 2.16, *p* = 0.151) or on the proportion of allogrooming interactions that lasted the full duration of a 30 s observation (F_1,58_ = 0.081, *p* = 0.777). The effect of immunization on allogrooming was therefore owing to the ants being more likely to allogroom, rather than being more likely to interact with the focal ant or allogrooming it for longer. Immunization had no effect on the frequency of self-grooming of non-treated ants (F_1,34_ = 0.04, *p* = 0.843).

**Figure 2.**
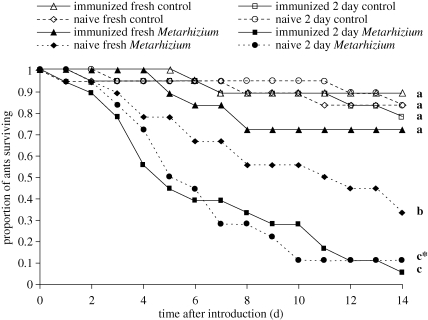
Survival of ants treated with either *Metarhizium* conidia or a control solution, and placed in either immunized or naive mini-nests either immediately or 2 days after treatment (*n* = 18). Letters indicate treatments that differed significantly at *p* < 0.05 (or *p* = 0.057 for b vs. c*) and also had *q* < 0.05.

Survival of control-treated ants was significantly greater than the survival of those treated with *Metarhizium* (Wald = 6.96, *p* = 0.008; figure 2). *Metarhizium*-treated ants introduced into mini-nests immediately after application survived better than those introduced 2 days after treatment (Wald = 3.82, *p* = 0.05), and ants placed in immunized mini-nests survived better than those placed in naive mini-nests (Wald = 4.3, *p* = 0.038). Although the interaction between these factors was marginally non-significant (Wald = 3.29, *p* = 0.07), pairwise comparisons indicate that the benefit of immunization was restricted to ants introduced immediately after treatment with *Metarhizium* (figure 2). Immunization did not affect the number of non-focal ants surviving at the end of the final assessment (mean ± s.e. per mini-nest: immunized = 15.7 ± 1.1; naive = 13.6 ± 1.4; *t*_34_=1.14, *p* = 0.261). None of the cadavers of control-treated and non-focal ants sporulated with *Metarhizium*, whereas 84 per cent of the *Metarhizium*-treated ants did so.

## 4. Discussion

The experiment showed that the social immune response depends on the nature of the threat (*Metarhizium* parasite or control) and whether the group has been previously immunized against it. Ants directed more allogrooming at ants exposed to the *Metarhizium* parasite than to those treated with the control solution. Allogrooming is effective at removing parasites such as *Metarhizium* from the cuticle and has previously been shown to be directed at individuals exposed to parasites (Rosengaus *et al*. 1998; Hughes *et al*. 2002; Yanagawa *et al*. 2008). Interestingly, however, we found that the ants tended to allogroom not only ants freshly treated with *Metarhizium*, but also ants treated 2 days previously, which were thus infected but not infectious. Ants may have been responding to cues left by *Metarhizium* on the cuticles of individuals treated 2 days previously or to their behaviour.

Allogrooming in response to ants treated with *Metarhizium* was significantly higher in mini-nests that had been immunized against the parasite than in naive mini-nests. Furthermore, this social immunization was effective, significantly improving the survival of ants freshly treated with the *Metarhizium* parasite. Levels of self-grooming did not differ, so the response does not appear to be a self-defence mechanism or a general increase in hygiene-related behaviour. Levels of antennation did not differ, so the response also does not appear to involve a general increase in activity or interaction rate. Rather, it appears that ants in immunized mini-nests were more likely to allogroom when they encountered an individual exposed to the parasite. Allogrooming of control ants did not differ between immunized and naive mini-nests, suggesting that the response was related to the *Metarhizium* parasite rather than simply to ant introduction or the carrier solution. In accord with previous work (Hughes *et al*. 2002), the survival of the non-focal ants was not affected by the parasite exposures (and thus did not differ between immunized and naive mini-nests), showing that the social immune response did not carry with it a cost of disease transmission.

The adaptive immune system of vertebrates is characterized by a memory of past parasite exposures, a heightened response to subsequent exposure, and the response being specific to the particular parasite previously experienced. This study has demonstrated the second of these three components in the social immune response of leaf-cutting ants. Further experiments will be needed to establish whether the other components are also present. However, the finding that the social immune response of *A*. *echinatior* is heightened by immunization reveals that it may be more complex than previously realized and may be functionally analogous to the advanced physiological immune response of vertebrates.
